# Optimizing Enteral Nutrition for Growth in Pediatric Chronic Kidney Disease (CKD)

**DOI:** 10.3389/fped.2018.00214

**Published:** 2018-08-02

**Authors:** Christina L. Nelms

**Affiliations:** ^1^PedsFeeds, Kearney, NE, United States; ^2^Department of Family Studies, University of Nebraska System, Kearney, NE, United States

**Keywords:** enteral, nutrition, pediatric, growth, renal, electrolyte, CKD

## Abstract

Growth in pediatric Chronic Kidney Disease is important for long-term outcomes including final adult height and cognitive function. However, there are many barriers for children with chronic kidney disease to achieve adequate nutritional intake to optimize growth. This review highlights these unique concerns, including route of nutrition, dialysis contributions and biochemical indices. Fitting the enteral feeding to the patients' needs involves choosing an appropriate product or products, limiting harmful nutrients in excess, notably aluminum, and altering for electrolyte and micronutrient needs. Unique adjustments to the enteral regimen include accommodating volume needs, optimizing macronutrient ratios, specific electrolyte adjustments, the blending of products together, and adjustments made to consider patient and family psychosocial needs. When a holistic approach to medical nutrition therapy is applied, taking the above factors into consideration, adequate intake for growth of the child with CKD is achievable.

## Introduction

Growth in chronic kidney disease (CKD) is a multifaceted clinical issue, complicated by physiologic linear height impairment, uremia, and frequently, developmental age lagging behind chronological age (1, 2). A unique aspect impacting growth in CKD is the need to tailor enteral feeding regimens in terms of route, formula type, and modulation of the enteral product to fit individual patient needs. Adjustments include accommodating dialysate volumes, variations based on type of dialysis, electrolyte management, gastrointestinal symptoms and psychosocial challenges common with CKD (1, 3). This review aims to highlight the specific challenges associated with optimizing enteral feeding to promote growth, especially in young children with CKD.

## Impact of nutrition on growth in CKD

The Kidney Disease Outcomes Quality Initiatives (KDOQI) pediatric nutrition guideline, considered a foundation for clinical nutrition care and practice for individuals with CKD, highlight several factors influencing the need for nutritional adjustment in children with CKD, including age, developmental stage, treatment or dialysis modality, residual kidney function, comorbidities, prematurity, decreased appetites or energy intake, acidosis, sodium losses, mineral bone disorders, and abnormalities in the growth hormone-insulin like growth factor axis (1, 2, 4). Poor growth has serious consequences, including hospitalization, mortality and poor quality of life (5). KDOQI specifically addresses the nutrition of young children, noting that spontaneous oral intake of less than 80% of estimated needs is common in infants and toddlers with CKD, worsening as the glomerular filtration rate (GFR) declines. In the peritoneal dialysis population, intake of <75% of energy needs is common as a result of a feeling of fullness from PD fluid, gastric emptying delays, variation in toxin removal and inability to achieve full dialysis prescription—thus compounding uremia. Even with some caloric intake from dialysis fluid, inadequate intake is common (6, 7). Correction of poor oral intake with supplemental feeds and tube feeding has been documented to improve growth and support catch up weight gain.

In the year 2000, Wong et al. (8) published data which greatly influenced nutritional goals within pediatric CKD. It was determined that for each standard deviation score (SDS) decline in children with CKD from healthy age appropriate norms, or SDS decline in growth velocity, a 14 or 12% respective increase in mortality was present, thus defining linear growth as more than a cosmetic issue. Additionally, mortality was noted to be in a U-shaped curve, with greater risks with very high or very low body mass indexes (BMI)'s. The North American Pediatric Renal Transplant Cooperative Study (NAPRTCS) data also shows poor survival with very short stature as well as poor outcomes when starting renal replacement if pre-dialysis linear growth is very poor (9). In 2016, Ku et al. (10) affirmed previous findings by Wong, also showing that poor height is correlated with reduced chance of kidney transplant. Children younger than age two often have the greater growth deficits and lower BMI's are noted for children under the age of five, while children older than five have higher BMI's, especially if CKD course was at an older age (11). However, children younger than age two also have the greatest chance of catch up growth while on dialysis (9). Enteral nutrition and supplemental feeding is a significant factor in these children's ability to meet adequate caloric intake and achieve catch up growth (12).

According to 2017 United States Renal Database Systems (USRDS) report data, over the past 10 years, children ages newborn-four have the highest prevalence of short stature, defined as <3% ile height or length for age, at 52.7%. Children ages newborn-four also have the highest incidence of being underweight, at 14.8%. Although nutrition alone is not enough to correct height deficiency, as the growth hormone-insulin like growth factor 1 (IGF-1) is typically impaired with CKD, correcting inadequate nutrition is the first step in ensuring linear growth can be optimized (13).

Children who are stunted or underweight are potentially at risk for protein-energy wasting (PEW). Once the cycle of inflammation and inadequate intake has begun, it is difficult to reverse. Children with protein energy wasting have high energy needs with concurrent anorexia and poor intake as well as significant breakdown of protein and muscle stores while being unable to utilize fat stores. Overfeeding only increases fat mass without promoting muscle regain (14). Prevention is ideal, however, clinical expertise is critical in determination of adequate but not excessive feedings to replete the patient with PEW to promote optimal growth. The International Society of Renal Nutrition and Metabolism (ISRNM) defined PEW in adult renal patients as “kidney disease wasting” specific to CKD or Acute Kidney Injury (AKI). Criteria includes low serum albumin, transthyretin or cholesterol, reduced body mass with reduced intake and reduced muscle mass. These criteria are used together for diagnosis as no single criterion alone meets the definition of PEW. The Chronic Kidney Disease in Children (CKiD) study (15), a large collaborative of children from North America with CKD who are not yet on dialysis, further defined these criteria in the context of pediatrics, including reference points which are pediatric specific, and also included poor growth in the diagnostic criteria, notably a height less than the third percentile or a decrease in growth velocity of more than 10%. This criteria is supported by general pediatric literature (16), including a joint position paper from the Academy of Nutrition and Dietetics (AND) and the American Society of Enteral and Parenteral Nutrition (ASPEN), noting that severe height stunting indicates repeat infections and/or chronic inadequate nutrition (17). The CKiD study revealed 7–20% of pediatric CKD patients have PEW (15). There is a paucity of data in pediatric dialysis patients regarding PEW. However, it stands to reason that since both appetite and weight declines are correlated with later stages of CKD, incidence of PEW is likely greater than what has been documented in earlier stages of CKD (18).

Although poor growth may be more common in young children with CKD, whose primary source of nutrition is breastmilk or formula, it is important to consider the types of foods promoted to children with CKD learning to expand solid food diets. Although caregivers may be anxious to offer whatever food the child is willing to take since oral feeding is physiologically delayed, encouraging varied healthful foods, to establish long-term appropriate eating patterns is necessary. CKiD data, including children that consume a solid food diet of 500 kcal or more, indicates that excess energy, protein and sodium is heavily present in children with CKD, with a high intake of fast food, salty snack foods and sugary foods such as candy contributing to the excess in caloric intake (19). The rates of obesity and overweight in all children with CKD are 18 and 15% respectively. BMI often declines after birth and then increases after age five (11), likely reflective of the shift from formula and enteral supplementation, to a solid food diet.

In a sentinel study by Coleman et al. (20) it was determined that children with gastrostomy buttons on dialysis received an average of 61% of their caloric needs via tube feeding. The same patients were able to achieve an average of 116% of the needs, promoting catch up growth. This demonstrates that tube feeding is typically necessary for young children with CKD, especially on dialysis, who require the support supplemental tube feeding provides. Gastrointestinal reflux is significant for children with CKD with severe occurrence 73% of the time (21), and up to a third of the feed may be lost to emesis (22). The need to compensate for these losses also supports use of supplemental enteral feeding in children with CKD.

Nutritional assessment for the youngest children, especially on dialysis, should occur more frequently than older children with earlier stages of CKD; up to weekly in age's newborn-one, receiving dialysis (1). Work by Coleman et al. (23), demonstrated that these youngest children needed assessment twice as often as older children with CKD, again, supporting up to weekly assessments with diet prescription being tailored to individual needs. Determining adequacy of growth and altering enteral feeding regimens can be done much more easily with such frequent assessment.

## Factors affecting enteral nutrition choices

Several factors affect enteral nutrition choices for children with CKD. As CKD progresses, the child becomes more uremic. This may affect appetite and increase gastrointestinal symptoms such as reflux and gastric emptying, creating challenges for meeting caloric needs. A child may need further supplementation or manipulation of enteral feeding products to supply enough nutrition. Growth may be compromised, especially as the child moves closer to ESRD. The clinician should be aware of kidney failure impact versus adequacy of nutritional intake on linear growth delays. When a child reaches the need for dialysis, the type of dialysis, residual renal function, and medications dictate the route and rate and what can be used for optimal enteral feeding. Original kidney disease impacts micronutrient and electrolyte needs (24). Monitoring growth and biochemical indices are the primary factors driving enteral feeding adjustments (3).

### Route of feeding

An important enteral nutrition choice is the route of feeding. Both nasogastric (NG) and gastrostomy tubes (G-tubes) are shown to promote adequate intake and catch up growth over demand feeding in pediatric CKD (25). Advantages of g-tubes include potential improvement of gastrointestinal symptoms, a more secure mode of feeding, without the need for frequent replacement of the tubing. Nasogastric tubes are more temporary and they require no surgical intervention. Disadvantages to nasogastric feedings, include increased gag reflux, frequent emesis, irritation to the back of the throat and nose, and associated aversions to future oral intake (26). Additionally, nasogastric tubes may be perceived as unattractive, and the risk for being pulled out or dislodged is high. Several studies have encouraged placement of gastrostomy buttons if it is anticipated that the child will require supplemental feedings longer than a few weeks. Children with CKD or on dialysis typically will need the support long-term. Early intervention with enteral feeding therapy supports catch up growth (12). This can be placed prior to or concurrently with the peritoneal dialysis (PD) catheter to decrease infection and peritonitis risk (20, 22).

Encouraging oral stimulation, including non-threatening use of food and gradual food introduction may aid with transition to a solid diet. Complete oral diet is typically delayed for children with CKD and may not happen until post-transplant or when the child is a few years old if remaining on dialysis (26). Transition to an oral diet is common after kidney transplant as most children without cognitive or developmental delay are able to meet caloric needs orally within 6 months post-transplant. Work with oral stimulation prior to transplant is helpful to expedite this progression (27). Pugh and Watson (28) recommend taking an individual approach and removing a feeding tube when the child is taking more than 50% of needs orally.

### Determining caloric needs for growth

When tailoring an enteral feed to the needs of a child with pediatric renal disease, the first step is to determine caloric requirements for growth. Basal metabolic rate (BMR) for children at stage 3 CKD have been shown to be comparable to healthy, age-matched children. BMR declines slightly as CKD advances. However, factors such as inflammation or comorbidities may increase caloric needs in later stages of CKD even if basal rates decreases. It is prudent to assume estimated energy requirement (EER) calculations, used with healthy children, are a good starting place to estimate needs and adjust as needed based on the child's growth (29). However, barriers such as appetite, uremia, and inflammation may impair the ability for children to receive the calories they need.

Continuous monitoring of growth and adjustment of the feeding prescription is pivotal to sustain adequate growth. Growth itself is a factor increasing energy demands, therefore enteral and/or oral solid feeding must be increased in tandem. Growth charts are an important tool for assessment and monitoring. The World Health Organization (30) charts should be used for children under the age of two and Centers for Disease Control (CDC) may be used after age two (1). Two important parameters are weight-for-length or BMI. Young children should maintain appropriate proportionality, and avoid declining weight in proportion to length or height. Although there are possible comorbid conditions or prematurity affecting head size in children with CKD, the head size growth curve should not decline. If no comorbidities are identified, a declining curve or an inadequately sized head could indicate poor caloric intake or chronic malnutrition. Short stature and decline in growth curve may be expected, and is influenced, by a variety of factors in CKD. However, decline in either weight-for-age or length/height-for-age should trigger assessment of nutritional adequacy and intervention, in the context of other CKD related factors. Likewise, rapid weight gain, or continued increase in weight-for-length or BMI not inclusive of catch up growth, can be an indicator of over-feeding (1). Average grams of weight gain per day can be an important clinical tool in the assessment and monitoring of nutrition adequacy. Tables with reference ranges of expected weight gain in grams per day are available from birth until age two (31). Standard deviation scores for both weight and height are also helpful clinical tools to trend growth (1).

### Dialysis

When CKD advances to end-stage, dialysis or transplant is necessary. With the additional fluid PD contributes, children often feel full, affecting the volume of enteral nutrition tolerated and possible gastrointestinal symptoms, such as reflux or emesis. Thus, continuous feedings during overnight dialysis may need to be adjusted to be lower in volume and higher in caloric density. Many children experience more symptoms the longer dialysis continues. Tube feeding may need to be limited in total volume, or stopped before dialysis is over.

The porousness of the peritoneal cavity and consequent ability to receive effective dialysis, known as “transport” status, can be classified as high, high-average, low and low-average and may change over the dialysis tenure. There are advantages and disadvantages to each regarding nutrition. Higher transport status ensures good solute removal; however, desirable nutrients, such as protein and potassium can be lost in greater amounts (32). Supplementation of protein and/or potassium in the enteral feed or as a medication may be indicated. Additionally, excess glucose absorption may occur (33). Carbohydrate from other sources may need to be limited. Overall, good solute removal may reduce symptoms of uremia and feeding may be better tolerated, allowing for more flexibility in volume of overnight feedings and the density of the feeding. On the opposite side of the spectrum, lower transporters typically do not lose as many beneficial nutrients across the membrane, but also do not receive the benefit of optimal solute removal. Consequently, less supplementation is needed and restriction of nutrients such as potassium may be necessary. However, due to uremia low transporters may also experience low tolerance for large volumes of enteral feeds. The clinician may need to plan for volume losses from emesis or make changes to administration of the feeding to promote tolerance (1).

Hemodialysis (HD) is less commonly used in young children with CKD, the population most likely to be receiving enteral tube feeding. Extracorporeal circuit volume needed as a percentage of blood volume poses a special challenge with the small size of young patients. Tight fluid control is necessary, limiting feeding options, especially in terms of volume. Hemodialysis is typically performed three times weekly, creating a need for tighter electrolyte control and use of a lower electrolyte formula may be indicated. Alternatively, some facilities increase hemodialysis to as often as six times per week for the youngest patients. Home hemodialysis is only available in a few centers in North America, but is another option to allow for liberalization of enteral feeding as typically blood pressure, clearance and serum potassium are improved with more frequent dialysis (34).

### Biochemical

Management of biochemical indices balanced with appropriate growth is paramount to good outcomes for the child with CKD. The clinician, ideally a pediatric renal dietitian, often must “cocktail” products together to provide a formula mixture appropriate for the individual child. This technique is fairly specific to the pediatric CKD population and requires a unique skill set. The KDOQI pediatric nutrition guideline outlines that enteral feeding and fluid intake often needs macronutrient, electrolyte, vitamin and mineral alterations specific to the needs of the child and their renal impairment (1). Electrolyte derangements have serious consequences for morbidity and mortality and thus, must be ordered as a first priority within goals for adequate growth. Other biochemical abnormalities may have less immediate, but still important implications for cardiovascular and bone health. Thus, biochemical indices are important factors when making enteral nutrition choices. Specific biochemical concerns will be discussed later on regarding fitting the enteral product to the patients' needs.

## Fitting enteral products to patient needs

Pediatric renal nutrition management involves several unique clinical factors making enteral product or formula choice more specialized. While typical children and even children with chronic medical issues, such as cardiac abnormalities, lung problems, failure to thrive, gastrointestinal (GI) issues, and others can use standard formulas or nearly standard formulas (such as lactose free, soy products, etc.), children with kidney disease often need formulas specific to managing biochemical abnormalities (1). Although standard products (i.e: regular infant formula, lactose free formula) may be used with some alterations there are several product choices specifically tailored for renal nutrition needs discussed below.

### Breastmilk

Breastmilk is known to be the optimal choice for almost all infants, with possible exceptions including rare genetic conditions or if the mother has an infection that can be spread through breastmilk, such as HIV. In the young child with CKD, breastmilk provides the same benefits to immunity, promotion of brain development, prevention of chronic diseases, psychological development, and other well-known general advantages documented in healthy children (30). However, there are some additional features especially ideal for CKD parameters. The bioavailability of breastmilk allows for ideal protein and micronutrient intake for the specific child's needs. Breastmilk is naturally lower in phosphorus and aluminum and is easily digestible because of its whey content, which is a major benefit for children prone to delayed gastric emptying, such as in CKD (35).

However, breastmilk is not always available for children with CKD. Maternal stresses, such as concern for medical issues related to CKD, prematurity or NICU stay, physical barriers such as tubing or medical devices limiting physical contact, affect the mothers' ability to feed at the breast. A mother under stress or who is physically away from her child may have trouble pumping enough breastmilk. Some mothers simply choose not to provide breastmilk for a variety of reasons. If breastmilk is available, it is almost always optimal to use for all of the enteral feeding, or as supplementation to a formula. Modulars, discussed below, can increase caloric density if needed. The clinician should do their best to work breastmilk into the feeding regimen, not wasting any of the typically hard-earned liquid, if the mother is willing and able to provide any, even for the short-term (36).

### Aluminum concerns

One well known concern with renal impairment is aluminum accumulation. Decades ago it was established that aluminum based products, especially aluminum phosphorus binders, were not safe for individuals with kidney disease, causing serious toxicity, mental impairment, and bone disease (1, 35). Excessive life-time aluminum accumulation should be avoided in children with kidney disease. Some aluminum accumulation may be unavoidable. For example, parenteral nutrition (PN) is a well-known source of unavoidable aluminum intake. Children who need PN will inevitably face aluminum intake. Medications may be another source of aluminum. Choosing medication alternatives that reduce aluminum intake and limiting PN as much as possible are ways to limit toxicity. Enteral formula also contains aluminum and formula choice can be a major factor in aluminum load the patient will receive. Hydrolyzed or elemental formulas are notable for aluminum content. Sometimes these formulas cannot be avoided when milk or soy protein intolerances or other GI issues dictate need. However, they should not be the first consideration if avoidable. Soy products are also much higher in aluminum content than other standard formulas and there is typically no advantage in use with children with CKD. Breast milk is the lowest in aluminum content, followed by whey-based formulas. Higher serum plasma levels of aluminum were seen in children who received soy or hydrolyzed formula compared to whey-based formulas or breastfed children. This is an important consideration when choosing the formula for a child with CKD (1, 35).

### Electrolytes and phosphorus

Children with CKD have specific electrolyte imbalances that make enteral product selection important. Although growth is a critical issue for long-term outcomes, electrolyte imbalances can have serious and sometimes deadly outcomes, thus making biochemical management the first priority (1).

A majority of infants and young children with CKD have disorders impairing the development of the kidneys and urinary tract. Other causes of kidney disease are common in pediatrics, but typically do not present until an older age (37). Sodium wasting is a common manifestation of physiologic changes in the kidneys and urinary tract. Children with these type of disorders, such as renal dysplasia, posterior urethral valves, reflux nephropathy, and others known as tubular disorders, are unable to reabsorb sodium in the distal renal tubule and consequently have great sodium and fluid losses. Adequate sodium intake is critical to neurological development and growth (38, 39). A formula or formula combination providing adequate sodium, or sodium supplementation through other means is necessary. Providing adequate fluid is also important to prevent dehydration and serious related consequences. However, there is a minority of young children with CKD who have original renal disorders that do not involve the sodium wasting seen in tubular disorders. Tight sodium control and fluid restriction to prevent the ill-effects of high blood pressure may be indicated. Children with tubular disorders on dialysis, with glomerular filtration rates (GFRs) low enough that urine output is impaired, may also need to limit fluid (1). Consequently, there is no one enteral product meeting the needs of all young children with CKD and a trained clinician must recognize these differences and recommend an appropriate enteral option.

Limitation of potassium is important as oftentimes children with CKD are unable to excrete potassium (40). Consequences of elevated or depressed serum potassium are serious, with the primary concern being cardiac arrthymia. Thus, many children with CKD also need an enteral formula or formula blend that limits potassium. A notable exception are children on peritoneal dialysis with very porous peritoneal membranes, known as “high transporters” (1). These children benefit from excellent uremic product removal, but may experience high protein and potassium losses.

Children with CKD may experience low serum bicarbonate levels. Low bicarbonate levels are known to impair linear growth and weight gain. Medication, such as sodium bicarbonate, can be given to patients either orally, via tube, or added to formula, to correct acidosis and improve growth (38). New options for improving alkali content using enteral feeding products will be discussed in the next section.

Dietary phosphorus restriction is not commonly prescribed in infants and children as the majority are primarily enterally tube fed, and most enteral products are low in phosphorus. In one study, children younger than age six were the only age group to have mean phosphorus levels within normal limits, attributed to use of low phosphorus enteral formulas. Thirty-eight percent of these patients were hypophosphatemic and phosphorus supplementation was appropriate (41). However, as the oral food diet advances for phosphorus restriction may become necessary. An elevated serum phosphorus has well known consequences for bone health and growth, and long-term implications for cardiovascular disease. Bone mineralization may be impaired as early as stage 2 CKD with abnormal bone turnover by stage 3 (42). “Decanting,” a method for removal of electrolytes from formula, described later in relationship to potassium modulation, can be used for phosphorus reduction, however this is seldom necessary as phosphorus intake from infant formulas rarely impacts serum values (43). If serum phosphorus levels continue to be elevated, despite dietary changes, phosphorus binders may be necessary. This is more commonly true as children begin to include more high phosphorus foods as solid food intake increases. Calcium based binders are considered the first choice for infants and young children (44), but non-calcium based binders may be used, especially in those with elevated serum calcium levels or for children who are exceeding twice the RDA for oral calcium intake. Aluminum based phosphorus binders are discouraged (1). A recent international, multi-center study (45) evaluated the use of sevelamer carbonate as a phosphorus binder in children aged birth through 18 and found it to be efficacious and safe in reducing serum phosphate levels. Children who received bolus tube feeding, but not continuous tube feeding were included in the study. Sevelamer carbonate was added to an appropriate amount of water as the administration method in applicable children. The recently published Kidney Disease–Improving Global Outcomes (KDIGO) guidelines suggest serum calcium levels influence phosphorus binder choice in children (46). Less commonly, calcium becomes a dietary concern. If a child needs multiple calcium based phosphorus binders, such as calcium carbonate, or has an above normal serum calcium, often related to vitamin D therapy, calcium may need to be limited in the oral diet and/or formula choice (44). If the child is not meeting the DRI for calcium through formula, diet, and calcium containing medications, calcium supplementation may be necessary, unless hypercalcemia is present (1). Again, because of the many factors that affect dietary sodium, potassium, phosphorus and calcium needs, a clinician must be skilled in product prescription.

### Vitamins and minerals

Finally, vitamin and mineral requirements may affect enteral nutrition needs (24). Children receiving dialysis treatments may experience losses of water soluble vitamins. Typically, these are replaced with a renal-focused multivitamin supplement. Fat soluble vitamins are not excreted appropriately in CKD, especially as GFR declines. Vitamin D has a unique role in CKD and usually active and inactive forms, are given at therapeutic levels, heavily medically managed, and thus outside the scope of enteral nutrition discussion. Vitamin E and vitamin K are known to be cleared by the kidneys and excessive intakes are not recommended for CKD, especially dialysis patients (1, 47). Importantly, vitamin A or serum retinol levels are known to be elevated in CKD. Excessive vitamin A has been linked to osteoclastic action in the bone as well as elevated serum calcium levels. The consequent elevated calcium levels are a risk for cardiovascular disease and further kidney damage (48). Thus, choosing enteral products that limit vitamin A is important, as children receiving a commercial enteral product are at highest risk for elevated retinol levels.

Controversy exists around the need for, and amount of vitamin and mineral supplementation. It is well known that water soluble vitamins are lost through dialysis (49, 50). However, the amounts lost may vary by the patient. Other medical comorbidities play a role as well. As noted above, vitamin K and E are fat soluble and excess accumulation is possible. However, children on multiple antibiotics could potentially experience vitamin K loss. Likewise, vitamin E is potentially not well cleared by dialysis, however, some data suggest that vitamin E may reduce oxidative stress in children at risk, or play a role in epogen-resistant anemia (51). Children on peritoneal dialysis who are high transporters may lose more vitamins and minerals compared to low transporters. Needs may also vary based on the type of renal replacement therapy (52). Some trace elements may be toxic if not monitored closely, while others may be deficient, especially for the child on dialysis (53). Zinc, especially is noted to be lost through dialysis and optimal amount of zinc supplementation is ideally tracked through assessment of serum levels (54). Excess magnesium levels have been reported and may be of concern (55). The KDOQI guidelines recommend a water soluble vitamin supplement or a “renal vitamin” for all children on dialysis (1). This may serve as an “insurance policy” for dialysis losses or poor intake. Sometimes, because of anorexia or similar issues, a renal vitamin may be started prior to dialysis in earlier CKD.

Children on formula supplementation are known to have higher vitamin levels (50, 56). Joyce et al. (57) recently looked at vitamin and mineral levels in children on dialysis. These children were typically on a renal, water soluble vitamin and some were on additional vitamins. Her work affirmed that these children almost always had high vitamin A levels, but also commonly had elevated vitamin B12 and vitamin E levels. Vitamin D levels were normal just over half of the time, but serum levels of other vitamins and minerals, such as folate, zinc, selenium, copper, and manganese might be elevated, normal or depressed. This highlights the challenge of meeting nutrient needs in children with CKD, as there are so many variables influencing choice of optimal enteral product or products. Whether the clinician should choose a product specific to the non-electrolyte vitamin and mineral content is controversial. Often the clinician has to rank or prioritize these variables. It also underscores the ongoing controversy regarding whether the enteral feeding should be supplemented with a vitamin/mineral product (50, 56).

## Unique adjustments to enteral feedings

A pediatric renal dietitian, experienced in managing young children and infants, must be knowledgeable and skilled in creating a unique enteral prescription to optimize growth while meeting micronutrient and electrolyte requirements.

### Volume restrictions

To achieve adequate growth, energy requirements must be met. This can be difficult if volume restriction is necessary, in the setting of little or no urine input, or inadequate dialysis. The pediatric renal nutrition professional should communicate closely with other members of the renal team to determine if volume allowance can or will be increased as dialysis increases or becomes more efficient. Volume restrictions may also be necessary to manage other physiologic factors. Children with CKD commonly have delayed gastric emptying or frequent emesis, spitting or gastroesophageal reflux (2, 21). Some children may have emesis or discomfort because they are on peritoneal dialysis and have large dextrose based “dwells” –the term used when peritoneal dialysis fluid is equilibrating in the body, removing toxins. The child feels very full and may not have much space or appetite for feedings. Thus, a clinician must adjust the volume or the timing of the feeds to minimize these issues since excessive emesis can reduce total nutrient intake. On the other hand, children with congenital kidney issues who lose water and salt may need enteral support. Some children are unable to tolerate a large volume of formula feeding, but still need free water to meet fluid needs. Timing of fluid intake is important to meet fluid needs but prevent emesis (22).

Adjusting timing of feeding or route of feeding may be one way to maximize volume. Offering continuous nocturnal feeds may alleviate emesis related to large day time bolus feeds. Likewise, increasing size or number of daytime bolus feeds could help alleviate emesis that occurs at the end of overnight feeds related to fullness (1). This is determined by individual assessment and trial and error with a given child.

Another way to optimize caloric intake is to increase the calories per volume amount. However, concentrating the formula and increasing calories also means concentrating electrolytes and other micronutrients that may be undesirable. Additionally, the renal solute load increases, compounding the burden on remaining kidney function (58). Choosing a “renal-friendly” product high in caloric density or use of modulars are strategies to optimize nutrient delivery. Careful monitoring for tolerance and management of related GI issues are important.

### Macronutrients

Macronutrient modulars are commonly used to concentrate the calories per amount of volume in the formulas and enteral products used for children with CKD (3). Children with CKD may need very specific amounts of protein. Limiting protein to the DRI may be important to delay the progression of CKD. Thus, due to small body size, protein intake is ideally very tightly limited in young children with CKD. However, once the child has reached dialysis, protein losses in hemodialysis and especially peritoneal dialysis increase those needs (1). Protein losses are twice as great per square meter of body surface area in infants on peritoneal dialysis as opposed to adolescent patients who are comparable to adults in size (59). Excess protein intake can increase body acid load which is especially problematic for bone health (60). Protein modulars can be used for children who require more protein and protein can be titrated to the individual patients' needs. Carbohydrate and fat modulars are commonly used to increase calorie concentration without significantly increasing micronutrients and electrolytes. Each has advantages and disadvantages. Carbohydrate modulars are simple to add and are typically well tolerated. However, excessive carbohydrate intake could increase triglyceride levels and, especially when a child is receiving dextrose from peritoneal dialysis, exceeding the acceptable macronutrient distribution range (AMDR) is a risk. Diarrhea is also a concern with excessive carbohydrate intake. Fat modulars, on the other hand, can help prevent excessive carbohydrate intake, especially when the child is also receiving glucose calories from PD, but also may be less well tolerated at least initially, and affect gastric emptying negatively. Typically, to maintain balance, fat and carbohydrates need to be increased in a balanced way (1).

### Potassium adjustments

Because many infants and children with CKD retain potassium, as noted above, limiting dietary intake is important (3). KDOQI guidelines recommend limiting potassium intake to 1–3 mmol/kg/day (1). Standard infant formula typically has 108–110 mg of potassium per 100 kcalories (kcal) based on a sampling of common standard infant formula (61–63). The only specific renal infant formula available in the United States market, Similac PM 60/40®, (64) has 80 mg of potassium per 100 kcal which is often too high for children with tubular disorders. There are a few options for reducing potassium intake in these children. One is using a product known as sodium polystyrene sulfonate (SPS) known by trade names kayexalate® or kionex® SPS is a sodium based resin and is often used as an oral medication, or in the case of extremely high serum potassium levels, rectal medication, that quickly reduces serum potassium levels. Because it has some gastrointestinal side effects, and in rare cases serious side effects such as bowel necrosis, in younger children with CKD it can be used instead to “decant” the formula. Through ion exchange, the sodium content of the SPS replaces potassium, effectively reducing serum potassium an average of 24% in 48 h (65). This addition of sodium to the formula may be beneficial for children who need sodium supplementation, but is not a benefit for children needing sodium limitation. Sodium content of formula increases on average 234% with this method (40). Calcium based resins, similar to SPS but with calcium instead of sodium are available in Canada but not the United States (66). The initial dose of SPS is often calculated by the pediatric renal dietitian. The recommended starting dose from the literature is 1 gram SPS per mEq of potassium (40), but in practice, many clinicians report starting with half this amount to prevent drastic serum potassium changes. The amount of SPS then can be adjusted based on subsequent serum potassium levels. The process involves treating the formula with the SPS for a half hour or longer, depending on facility protocol, and then carefully transferring the potassium-reduced formula to a new container. The bottom of the old container will contain a “sludge” like substance where the bound potassium is concentrated and this is discarded. Although a very popular practice in North America, this practice is rarely considered in parts of the world that have more infant and pediatric formula options. Use of SPS alters other nutrients, typically lowering amounts, including calcium, copper, manganese, phosphorus, magnesium and zinc. Some formulas' nutrient profile is greatly altered by the SPS, others are not. This make it difficult to know what nutrients the individual is receiving and whether micronutrient inadequacy or excess may be occurring. Additionally, use of a liquid suspension greatly increases aluminum content of the formula (36). Thus, if this product is used, the powdered version should be chosen. Although considered to be safer than oral or rectal administration, there are still risks with the decanting process, including common risks of serious biochemical derangement (67) as it is not possible to remove all of the SPS solution from the decanted formula.

Another technique used to reduce potassium intake of young children with CKD is to use ready-to-feed adult-based renal formulas (68). Suplena®, (69) is most commonly used as its protein profile, as an adult pre-dialysis product, is more consistent with the protein needs of young children on dialysis. The high calorie to potassium ratio, as most adult products are concentrated to allow for fluid restrictions common to dialysis, is a means to lower potassium intake while achieving adequate caloric intake for growth. However, reports of gastrointestinal intolerance have been reported with this practice and the micronutrient profile is not designed for a pediatric population (65). Thus, vitamin and mineral excesses are possible.

Lastly, there are products on the market that are not intended to be used as sole source nutrition, but are very low in specific electrolytes and micronutrients, thus making them appropriate to modulate existing infant or pediatric products. These products have often been used standardly in other parts of the world, but are now gaining popularity in the U.S. market. Renastart®, a specific pediatric renal product is low in calcium, protein, chloride, phosphorus, potassium, and vitamin A (70) is often used to mix with other enteral products to reduce the overall potassium content of the formula (3). Renastart® is indicated for ages one and older in the U.S. but is indicated as an infant formula in other parts of the world. Renalcal® is an adult product that is also not a stand-alone product, significantly low in most micronutrients and electrolytes, and must be used cautiously in children who are getting no or little oral nutrition outside of formula (71). It also can reduce the total potassium content of a formula when it is mixed with other enteral products.

### Sodium

Providing adequate sodium is important for electrolyte management and neurological concerns and directly impacts growth as well (1, 38, 72). As it is easier to add nutrients as opposed to removing them, providing additional sodium is often considered easier than limiting potassium. Supplementation of 2–4 mEq of sodium per 100 mL of formula is recommended. Sodium can be increased in the enteral product by adding an amount of sodium chloride, sodium bicarbonate or similar product to the formula (73). Some children do not like the taste of this addition, but directly supplying sodium preparation through a feeding tube, flushed with water afterward is another option (22). As most infant and pediatric formulas are low in sodium (72), another option is adding a product such as Renastart® (70). Designed as a renal product for children with renal tubular disorders, Renastart® is higher in sodium. As a child starts an oral solid diet, providing adequate sodium is usually much easier. Many children have an affinity for salty foods and even a small amount of oral intake, with a “western diet” –known for being high in salt, can meet these additional needs for sodium. However, high sodium foods should not be encouraged to become regular parts of the diet as oral intake increases, risks for hypertension and fluid retention occur with excess (1, 19).

### Bicarbonate

Correction of serum bicarbonate (CO_2_) levels to at least 22 mmol/L is recommended (13) to improve growth and slow potential decline to ESKD (74). However, two-thirds of children who are acidotic (serum CO_2_ <18 mmol/L) are not on alkali supplements (11). Formulas like Renastart® (70) and Renalcal® (71), that are specifically low in content of certain nutrients such as chloride, can modulate existing formula to reduce the overall chloride content, possibly improving acid-base profile. These newer products are the only known formulas created to limit chloride and are both used in supplement with another source of nutrition.

### Creating unique feedings

In most pediatric disorders that involve risks for growth failure, a single enteral nutrition product is used with minimal adjustment. However, pediatric renal patients often benefit from a “cocktailing” of products. Adjustments can be made by mixing products together and adding modulars to tailor the individual formula prescription to the growth and biochemical needs of the child. The biochemical and electrolyte concerns drive these product combinations. Occasionally, choosing a formula that is easy to digest, such as a product high in whey protein, is necessary to work around issues with delayed gastric emptying or gastrointestinal reflux.

A potential solution is providing real-food blended tube feedings. Unlike the mixing of commercially available products noted above, this involves using foods blended to create a unique and specific enteral formula product. The advantage of this technique, seen by many clinicians and families, is the introduction of real, physiologic food, which potentially has beneficial effects on gut flora and synergistic effects of real food intake. Sometimes commercially available formulas or modulars are used as part of the real-food formula to help meet nutrition needs. If a clinician chooses this route, they can design a formula that meets kcal needs while matching electrolyte restrictions (75). Food safety and proper delivery of the enteral formula is essential when using real food products and may not be appropriate for every family who is unwilling or unable to go to the extent of necessary food safety measures or tale the time needed to create homemade formula.

A specific challenge for the pediatric renal team is the child with a milk or milk-soy protein allergy or severe intolerance. None of the formulas designed for renal needs are adapted to be milk and/or soy free (63, 70, 71). Children with severe intolerances can have major gastrointestinal issues, including severe vomiting or blood in the stools. Some children may have additional GI issues or could have a secondary condition such as short-gut or mal-absorptive disorders. In these instances a hydrolyzed formula may be necessary. Although these are certainly not ideal for kidney disease because of micronutrient profile as well as the higher aluminum content of hydrolyzed formulas, the child must receive adequate nutrition for growth (35). Thus, managing electrolytes through other means, such as use of SPS to treat the formula, phosphorus binders, and additional sodium may be necessary. The renal clinician must carefully follow laboratory trends and growth to make adjustments, creating the best possible clinical situation for the child. Sometimes prioritizing is necessary. Development of a product to meet the needs of children with these intolerances would address this specific gap in care and is an area for industry and research to consider.

Formula choice may vary greatly across the world. In North America, the aforementioned Similac PM 60/40® (64) is the only available infant-specific formula for CKD with products like Suplena® (69), Gerber Good Start® (62), Renastart® (70) and Renalcal® (71) being used as needed, often to modulate existing formula. Nephea Kid® (76), similar to Renastart® (70), is available in Canada and parts of Europe. However, in Europe and Australia, Kindergen® (77) is a product that is marketed for CKD, especially for PD patients. Occasionally, for specific renal impairments, a low calcium formula such as Locasol® (Europe) (78) or Calcilo XD® (North America) (79) is used. Oftentimes regular infant formulas are used and are modulated with other formulas or adjustments made using phosphorus binders, SPS, sodium or other mixtures. In some countries, specialized products are not available. There are a variety of clinical and social scenarios in which a variety of formula choices may be made, some of which may not be specific “renal formulas.” Again, the skilled clinician must just consider management of macro and micronutrient needs, electrolytes and other biochemical indices and, importantly, growth and development, when adapting the feeding prescription. Table 1 highlights common formulas used in the United States, specific to renal implications and includes information on important nutrients and other factors to consider.

**Table 1:** Standard nutrient content of products commonly used in children with CKD − nutrients significant for CKD.

**(per 100 kcal)**	Similac Advance®^*^	**Breastmilk, mature term**	**Similac PM 60/40®**	**Gerber Good Start Gentle®**	**Suplena®**	**Renastart®**	**Renalcal®**
Protein	2.07 g	1.35	2.2 g	2.2 g	2.5 g	1.6 g	1.7 g
Sodium	25 mg	29 mg	24 mg	27 mg	45 mg	50 mg	3 mg
Potassium	110 mg	72 mg	80 mg	108 mg	64 mg	23 mg	4 mg
Phosphorus	44 mg	20 mg	28 mg	38 mg	40 mg	19 mg	5 mg
Calcium	82 mg	35 mg	56 mg	67 mg	59 mg	22 mg	3 mg
Magnesium	6 mg	4 mg	6 mg	7 mg	12 mg	10 mg	1 mg
Vitamin A	300 IU	302 IU	300 IU	300 IU	176 IU	91 IU	0 mg
Vitamin D	75 IU	4 IU	60 IU	75 IU	5 IU	42 IU	0 IU
Vitamin E	1.5 IU	0.2 IU	1.5 IU	2 IU	5 IU	0.9 IU	0 IU
Vitamin K	8 mcg	4 mcg	8 mcg	8 mcg	5 mcg	6 mcg	0 mcg
Other notes	For comparison, standard infant formula	First choice for CKD if available due to bioavailability and other beneficial properties of breastmilk	60% whey	100% whey, not renal-specific	1.8 kcal/mL, adult pre-dialysis, casein based has fiber	pediatric, very low Cl-„ K+, Ca++, 100% whey	2 kcal/mL, adult product, very low mineral, 100% whey

* *Similac advance included only for purposes of a baseline comparison to a regular infant formula. (31, 62, 63, 70, 71, 80)*.

### Socioeconomic factors

One final challenge for the pediatric renal clinician involves working with families of all skill levels, financial means and abilities. A dietitian can create the best possible titration of formula composition, with modulars and specialty formulas to meet a child's growth, biochemical, and individual macro and micronutrient needs. However, if the child does not receive this formula or the entirety of the formula, its specifications are inconsequential. Families of young children with CKD may have large emotional and cognitive burdens. Worrying about a sick child, learning to do dialysis or other medical cares, stresses of time spent in the hospital and away from home or work, learning multiple medication needs, and much more, leave little room for mixing and administering formula (81). In fact, many parents say that frustrations surrounding the lack of their child's ability to eat “normally” is one of the most overwhelming parts of having a child with CKD or on dialysis (28). Parents have to learn regimens for providing tube feedings to their child, encouraging some oral intake, often coping with emesis, reflux or other gastrointestinal concerns, as well as issues like acute illness that may make feeding even more difficult. Multiple steps to prepare the formula is often more than families can cope with. Some parents have limited literacy skills, or difficulty following multiple instructions. Some parents are single parents or have other children's needs to care for. Some parents simply can become confused with the multiple cares they are responsible for providing; and mistakes in formula mixing, remembering instructions, or less-than-ideal formula mixing conditions are realities that the clinician should plan and evaluate for without judgment.

A clinician must evaluate, with careful inclusion and input from the family, the benefits of creating a formula mixture that is optimal for the child's nutrition, growth and biochemical needs, versus the risks that the family may not be able to complete and provide that regimen. Although providing an appropriate formula is always crucial, compromises may need to be made to fit the families' needs. For example, many families benefit from using liquid formula, or liquid formula mixed perhaps only with one or two other specialty products as they can open the liquid formula and just pour it in the feeding or mixing container. Limiting the number of specialty products or frequency of formula changes or steps in the change may be necessary. Sometimes the skilled clinician must be content to monitor biochemical indices to determine if a formula that fits the families' needs, but may not be “perfect” may be adequate if it does not cause significant harm to the patient (82). A need for future development in this field is the potential creation or availability of products that meet specific needs of this population, but with easy mixing and/or use to reduce chance for human error.

Additionally, financial challenges may provide a barrier. The pediatric renal dietitian must work closely with social workers, nursing staff, and outside resources such as community and government programs, to make it possible for families to afford the formula their child needs. Sometimes a product that does allow for growth and normal electrolytes may be necessary, even if not an ideal macro or micronutrient profile, if it prevents undue stress on a families' resources. For this reason the product Gerber Good Start® (62), is sometimes used, especially earlier in CKD, as it is a general infant formula and does not cost as much as specialty formulas. Its whey content and lower phosphorus levels make it a possible option.

### Pros and cons to consider

Enteral product choice will always be very individual, based on patient specifics. Breastmilk is always an ideal option if it is available (1). If volume limitations are the priority, more concentrated products, such as Suplena® or Renalcal® or concentrated Renastart® may be better options as well as the use of modulars. If gastrointestinal issues are the primary concern, a whey based formula and a lower density formula should be considered, such as Similac PM 60/40®, a standard whey based formula that has been modified to meet renal needs, or a diluted Renastart®. A specifically tailored real-food blenderized product can be considered. If socioeconomic factors, or risks of contamination are more in the forefront, an easy-to-mix, liquid product, such as Suplena®, or Renalcal® may be more appropriate. The number of steps involved in making the formula should be considered. If biochemical indices are of the greatest concern, choosing a product to meet those indices (i.e: Renastart® or Renalcal® for management of acidosis and potassium, Renastart® for adequate sodium intake, Similac PM 60/40® or Suplena® over a general product). Limitations should be considered as well. Some facilities do not allow the use of powdered products due to contamination risk (83). Renalcal® and Suplena® are designed for adults and have limitations for use in children. The clinician must decide which factors are most important when making these choices. Pros and cons of timing of feeding and nocturnal, continuous and bolus feeding must also be decided.

## Recommendations and conclusions

The clinician who is involved with young children with CKD must prepare themselves for the challenges of enteral feeding. They must seek out the training to understand the complexities of electrolyte abnormalities, pediatric-specific biochemical ranges, growth expectations, tracking growth closely and products available to meet the child's needs.

The clinician must first determine route of feeding, caloric needs for growth, and then select the product or products and amounts that will achieve these caloric goals, determine rates and times the feeding is to be given, and adjust the feeding to meet the unique needs discussed throughout this review, using modulars/additives, medications, and supplements as needed.

Specifically, a trained, pediatric renal dietitian is the best person to recommend enteral feeding regimen for a child with CKD (33). This clinician must understand the primary renal disease and associated electrolyte challenges, notably with sodium, potassium, and phosphorus. The clinician must become familiar with micronutrient adequate intakes (AI's) and tolerable upper limits (UL's) for the ages they are working with as well as recommended macronutrient intakes. They must be aware of concerns of excess aluminum intake and gastrointestinal challenges children with CKD face. Lastly, those determining formula prescription must be aware of the products available to them and the pros and cons of each as well as the social and financial barriers of families that play into those choices. See Table 2 for a summary of enteral feeding adjustments that may need to be considered.

**Table 2:** Factors affecting enteral feeding choices to optimize growth in pediatric CKD.

Growth	Meeting growth chart and monthly weight gain expectations in grams; assessing for emesis and adequacy of feeds
Biochemical	Assessment of electrolytes for adjustment to feeds; changing an enteral product, adding medication such as SPS or sodium bicarbonate, modulating or mixing of enteral products
Route	If any sign of poor intake, consider placement of gastrostomy, gastrostomy preferred for later transition to oral diet; typically difficulty meeting oral intake needs spontaneously; consider use and adjustment of continuous and bolus feeds to meet child's individual tolerance needs
Renal Replacement Therapy	CKD patients may need to limit protein, have more freedom with electrolytes; hemodialysis must tightly limit electrolytes typically, PD patients may have needs that depend on transport status – such as varying needs for protein, potassium, etc.
Original Kidney Disease	Renal tubular disorders typically involve high sodium needs, tight potassium needs and fluid loss, while other conditions typically require stricter electrolyte control overall
Macro and Micronutrients	Balance of macronutrients is ideal, titrating for protein needs; micronutrients should meet DRI standards while avoiding exceeding UL's; specific micronutrients may be in abundance or shortage in CKD; close attention to electrolytes and other biochemical indices is necessary
Comorbidities	Considerations include gastrointestinal impairment, need for hydrolyzed or milk-soy free formula, other organ involvement; manifestations of specific original kidney disease
Volume concerns	Children with tubular disorders may need adequate fluid intake due to high volume losses; other children may need tight limitations, or have to limit fluid as urine output declines; factors such as emesis and gastric emptying may alter rate and times which fluid may be given
Psychosocial	Family and patient challenges as to complexity of formula and feeding regimen must be addressed with consideration of educational, financial, literacy and other needs, including family stressors and burdens

Specific gaps in care and opportunities for development in the field include a need for products to simplify the mixing of enteral feeds, as well as meet the needs of children with milk and/or soy intolerances or other GI needs. This may enable more children to meet full growth potential by ensuring adherence and adequacy of feeding.

Growth in early childhood is essential for final height potential, cognitive development and psychosocial development. Balancing all considerations and optimizing the enteral feeding regimen is key for growth in pediatric CKD.

## Author contributions

The author confirms being the sole contributor of this work and approved it for publication.

### Conflict of interest statement

The author declares that she is an independent consultant and educator in the field of pediatric renal nutrition. She previously worked at pediatric facilities specializing in renal nutrition, primarily Children's Mercy Hospitals and Clinics in Kansas City, MO. She currently has contracts with Vitaflo, USA, and Fresenius, North America. Work on this manuscript was completed independently.
